# Patients with tinnitus use more primary healthcare compared to people without tinnitus

**DOI:** 10.1038/s41598-021-96607-x

**Published:** 2021-08-27

**Authors:** Maaike Maartje Rademaker, Inge Stegeman, Mariette Hooiveld, Robert Jan Stokroos, Adriana Leni Smit

**Affiliations:** 1 Department of Otorhinolaryngology - Head and Neck Surgery, University Medical Center Utrecht, Utrecht, The Netherlands; 2 University Medical Center Utrecht Brain Center, Utrecht University, Utrecht, The Netherlands; 3 Department of Ophthalmology, University Medical Center Utrecht, Utrecht, The Netherlands; 4 Epidemiology and Data Science, Amsterdam University Medical Centers, University of Amsterdam, Amsterdam, The Netherlands; 5 Nivel (Netherlands Institute for Health Services Research), Utrecht, The Netherlands

**Keywords:** Diseases, Neurological disorders, Health services, Epidemiology, Neurology

## Abstract

Tinnitus is a heterogeneous condition not only in terms of nature of the sound, but also in co-morbidities such as mental health issues. Prevalence number range widely between 5 and 43%. Even though the etiologic pathway between tinnitus and its comorbidities remains unclear, in this study we aim to assess whether people with tinnitus use more primary health care than people without tinnitus. To compare primary healthcare consumption between patients with tinnitus and people without tinnitus. In this cross-sectional study, data on number of consultations with the general practitioner or nurse practitioner mental health services were obtained from Nivel (Netherlands Institute for Health Service Research) Primary Care Database in 2018 (n = 963,880 people). People with an open tinnitus episode (n = 8050) were defined as a patient with tinnitus and compared to all other people. Percentages, means, ranges and mean differences were calculated for the total number of consultations and for organ specific diagnoses registered as ICPC-1 code on the day of consultation. Secondary, the total number of referrals to medical specialists and number of drug prescriptions was collected. Logistic regressions were performed to predict having one or more contacts, referrals, and prescriptions,with having tinnitus, this was corrected for age and gender. Patients with tinnitus had a mean of 9.8 (SD 10.9) primary care consultations in 2018, compared to 5.7 (SD 7.9) for people without tinnitus. More patients with tinnitus had more than one referral to medical specialists (47%) compared to people without tinnitus (25%). Patients with tinnitus have 1.2 (mean difference) more drug prescriptions than people without tinnitus. Compared to people without tinnitus, patients with tinnitus were more likely to have one or more of primary healthcare contact, independent of age group and gender. Patients with tinnitus had more consultations in primary health care than people without tinnitus. They are more often referred to medical specialists and receive more drug prescriptions. The causal relationship between tinnitus and the higher healthcare consumption remains to be researched.

## Introduction

People with tinnitus perceive a phantom sound, in absence of an external stimulus^1^. It is a complex condition that affects around 5 to 43% of the population^2,3^. Tinnitus does not only vary in terms of nature of the sound, location and pitch, but also in hindrance in a person’s life^3^. Whilst some are not bothered by the tinnitus, for others it can severely impair daily life. Reported numbers indicate that up to 5% of the population are mildly to moderately disturbed by their tinnitus^4–6^. Quality of life is described to be severely reduced in 1–2% of tinnitus patients^7^.

Currently, a cure for tinnitus remains to be found. Treatment is focused on symptom reduction. Many treatments are available, however at this moment only Cognitive Behavioral Therapy (CBT) has been proven to diminish tinnitus distress^8^. Other possible treatment options for tinnitus patients with hearing loss includes hearing aids or sound therapy^9^.

In the European guideline for tinnitus health care a stepwise approach for tinnitus care is proposed^10^. In this approach, the general practitioner (GP) is advised to screen for hearing loss and bothersome tinnitus. In case of bothersome tinnitus a referral to an ENT surgeon and/or audiologist is indicated for diagnosis, assessment of the tinnitus severity and to facilitate counselling, education and hearing rehabilitation when necessary. If these steps are not sufficient, specialist tinnitus healthcare is recommended. This includes psycho-education and/or CBT^10^.

The socio-economic costs of tinnitus are considerable, due to the high prevalence, and the chronic nature of the condition^11^. Also, patients often undergo multiple different treatments^12^. In the Netherlands, the mean societal cost (healthcare costs, patient and family costs & indirect costs) of tinnitus are estimated at €6.8 billion per year, with a mean of €1.9 billion for health care costs alone^13^. In the United States annual healthcare costs for tinnitus are estimated at $660 per patient per year^14^. A recent analysis of the treatment costs for tinnitus in Great Britain resulted in an estimation of £750 million per year^11^. So far, it remains unknown if patient or tinnitus related characteristics or comorbidities influence the health consumption of people with tinnitus. This can be of importance since health care costs are incremental and the ongoing debates about the cost-effectiveness of offered experimental and non-experimental therapies^11^.

So far, different comorbidities have been reported to be associated with tinnitus. For example, in a US cohort of the general population, 26% of the tinnitus patients reported anxiety problems, 26% reported depression and people with tinnitus reported significantly fewer hours of sleep per night compared to people without tinnitus^15^. Other studies showed that individuals with tinnitus more often encounter physical problems compared to individuals without tinnitus^16^. In a recent systematic review including 55 studies, multiple significant associations between non-otological risk factors and tinnitus presence were described. These included psychological factors, demographics, musculoskeletal and cardiovascular factors^17^.

Not all people that are aware of tinnitus sounds experience “emotional distress, cognitive dysfunction, or autonomic arousal, leading to behavioural changes and functional disability”^18 p8^. So far, it remains unknown when a person with tinnitus becomes a person with tinnitus disorder^18^. It would be interesting to look at the role that co-morbidities play in this. We wonder whether tinnitus patients do not only seek help for tinnitus, but are in need of more health care in general. This is of interest since the relation between tinnitus and its co-morbidities remains a story of ”the chicken and the egg?” Does tinnitus make people prone for other diseases? Or is it vice-versa and do other diseases make people prone for tinnitus? Even though this question will not be answered in this study, we will take a first step in assessing the differences.

In this paper, we study the differences in health care consumption between patients with tinnitus and patients without tinnitus in primary care in a cross-sectional study.

## Methods

This paper was written according to the Strengthening the Reporting of Observational Studies in Epidemiology (STROBE) statement^19^.

### Study aim and design

In this cross-sectional study our primary objective is to asses differences in primary health care consumption of people with and without tinnitus.

### Study population

The sample for this study was taken from the participants to the Nivel primary care database^20^. This is national representative longitudinal cohort of which systematical health care consumption data is registered out of the electronic health care records of Dutch primary care health care professionals.

For this study we used data derived out of electronic health records of 295 general practices contributing to the Nivel primary care database from 2018. Data was collected on the total number of consultations to the GP or mental service nurse practitioner [‘’Praktijkondersteuner Huisarts—Geestelijke Gezondheidzorg’’ (‘’POH-GGZ’’]. This was based on claims to health care insurances. In the Netherlands, all non-institutionalized inhabitants are compulsorily enlisted with a general practice, including patients who do not visit their GP on a regular basis. The GP is the first professional to consult for health problems and has a gatekeeper role for specialized care. Therefore, our data included all enlisted persons, including those that did not contact the GP or mental service nurse practitioner in 2018.

### Outcomes

The data included details about total number of consultations (consultation at the practice, at home, by phone or email). Demographic data were collected including age group of the participants (18–39, 40–64, 65 + (determined at 31st of December 2018) and gender.

Diagnoses in primary health care are registered according to the International Classification of Primary Care (ICPC version 1)^21^. For each consultation, a maximum of three different diagnosis codes registered on the same day were linked.

ICPC codes are organized in 17 individual chapters based on body systems representing the localization of the health problem. Of these 17 chapters, we combined the chapters with psychological and social problems into psychosocial problems. All other individual chapters of the 17, except for the psychological and social problems were combined into ‘other’. If no diagnosis code was registered on the day of consultation, the consult was categorized as diagnosis unknown. Theoretically, one would expect 0 contacts for males in the ICPC code reproductive organs for females and vice-versa. However, this is not the case. For example, it could be that a contact was registered because a male had a question on female reproductive organ. For this reason the mean for gender specific ICPC codes (reproductive organs male/female and pregnancy) were only reported for males or females respectively.

Next, the number of different drug prescriptions (anatomical therapeutic chemical (ATC) classification level 3) were collected^22^. For a subset of 113 general practices, data on referrals to medical specialists were available. We obtained the number of referrals to different medical specialists. Multiple referrals to the same medical specialty were counted as one.

To obtain data about the number of patients with tinnitus, we looked at prevalent cases of tinnitus in 2018 with an open diseases episode of tinnitus^23^. The open tinnitus episode was defined as a registration of tinnitus diagnosis in the patient’s electronic health record within the period mid-2017 to the end of 2018^23^.

### Ethical considerations

We obtained permission from the Nivel steering committee (with representatives from national associations of general practitioners) to use the data (as presented in this study), from the Nivel Primary Care Database. This study has been approved according to the governance code of Nivel Primary Care Database, under number NZR-00318.048. The use of the electronic health records for research purposes is allowed under certain conditions. When these conditions are fulfilled, neither obtaining informed consent from patients nor approval by a medical ethics committee is obligatory for this type of observational studies containing no directly identifiable data (art. 24 GDPR Implementation act Jo art.9.2. sub).

### Statistical analysis

Statistical analyses were performed with SPSS version 25.0.0.2. Count data was reported with means, standard deviations and ranges. Frequencies and percentages were presented for 0 counts and > 1 counts. Due to the nature of the data (count-data) and the largeness of the dataset we did not check for normality^24,25^. Subgroup analyses between people with and without tinnitus were performed. Statistical significance is easily reached in large datasets^26^. We therefore did not assess statistical significance between both groups. We calculated mean differences and 95% confidence intervals (CI) between subgroups. Based on expert opinion we considered a mean difference of 1 visit to be clinically relevant for the number of drug prescriptions between groups. A mean difference of 2 was considered clinically relevant for differences in the total number of consultations between groups because patients with tinnitus are expected to have at least 1 extra consultation for their tinnitus diagnosis. We considered a mean difference of 1 between groups to be clinically relevant for the different ICPC codes. The data has a hierarchical nature, based on general practices. Since we used all 295 general practices in the analyses and because of the largeness and representability of the sample we did not use multilevel regressions. We categorized the variables: total amount of visits, referrals and prescpritions into two categories (having either 0 or ≥ 1 visits, referrals or prescriptions). We performed complete-case binary logistic regressions to assess the influence of having tinnitus on having ≥ 1 visit, referral or prescriptions. These binary logistic regressions were corrected for gender and age, since older people are more likely to use more care. We checked for multicollineairty.

## Results

Data about health care consumption of 963,880 individual people were collected. 488,629 were female (51.1%). 314.421 people were 18–39 years old (32.6%), 412.842 were 40–64 years old (42.8%) and 136.617 were 65 years or older (24.5%). 8050 people (0.8%) were defined as patients having tinnitus. More males (n = 4189, 52%) than females (n = 3861, 48%) were defined as a patient with tinnitus. 6500 of 8050 patients with tinnitus (80.7%) were 40 years or older, while 642,959 of 955,830 (67.2%) of the people without tinnitus were 40 years or older. (Table 1).

**Table 1:** Gender, age and number of referrals of patients with and without tinnitus.

	Total number of patients (*N *(*%*))	Tinnitus	
		No (n = 955,830) (*N *(*%*))	Yes (n = 8050) (*N *(*%*))
**Gender**			
Male	471,390 (48.9)	467,201 (48.9)	4189 (52.0)
Female	492,490 (51.1)	488,629 (51.1)	3861 (48.0)
**Age (years)**			
18–39	314,421 (32.6)	312,871 (32.7)	1550 (19.3)
40–64	412,842 (42.8)	408,572 (42.7)	4270 (53.0)
65 +	236,617 (24.5)	234,387 (24.5)	2230 (27.7)

### Number of primary care contacts

The total mean number of primary care contacts for the complete sample was 5.77 (SD 7.97) in the year 2018 (range 0–338). 762,504 (79.1%) of 963,880 had 1 or more primary care contacts in 2018. The total number of primary care contacts was higher in patients with tinnitus compared to people without tinnitus (mean difference 4.03 (95% CI 3.85–4.19). 754,815 of 955,830 (79%) had one or more primary care contact, compared to 7689 of 8050 patients with tinnitus (95.5%) Within these, patients with tinnitus had a mean of 1.13 more contacts related to the ICPC code ear (95% CI 1.10–1.17) compared to people without tinnitus. Patients with tinnitus had a mean of 0.48 (95% CI 0.41–0.55) more contacts related to psychological symptoms or diagnoses, and a mean of 0.44 (95% CI 0.39–0.49) more contacts for musculoskeletal contacts compared to people without tinnitus. Consultations for all organ system but pregnancy, were more frequent in patients with tinnitus. (Table 2).

**Table 2:** Number of different diagnoses per ICPC code.

ICPC codes	Total (n = 963,880)	Tinnitus		Mean difference (95% confidence interval)
		No (n = 955,830)	Yes (n = 8050)	
**Total consultations**				
(Mean (SD) (range)	5.77 (7.97) (0–338)	5.74 (7.93) (0–229)	9.76 (10.88) (0–338)	4.03 (3.79–4.26)
≥ 1	762,504 (79.1)	754,815 (79.0)	7689 (95.5)	
**General**				
(Mean (SD) (range)	0.49 (1.46) ( 0–102)	0.49 (1.46) (0–102)	0.69 (1.82) ( 0–61)	0.20 (0.16–0.24)
≥ 1	216,010 (22.4)	213,591 (22.3)	2419 (30.0)	
**Musculoskeletal system**				
(Mean (SD) (range)	0.79 (1.79) (0–73)	0.79 (1.79) (0–73)	1.23 (2.29) (0–29)	0.44 (0.39–0.49)
≥ 1	312,132 (32.4)	308,526 (32.3)	3606 (44.8)	
**Blood**				
(Mean (SD) (range)	0.12 (0.97) (0–124)	0.12 (0.97) (0–124)	0.18 (1.24) (0–40)	0.07 (0.04–0.09)
≥ 1	35,878 (3.7)	35,449 (3.7)	429 (5.3)	
**Endocrine glands**				
(Mean (SD) (range)	0.35 (1.34) (0–101)	0.35 (1.34) (0–101)	0.52 (1.75) (0–54)	0.17 (0.14–0.21)
≥ 1	129,141 (13.4)	127,611 (13.4)	1530 (19.0)	
**Reproductive organs (male)**^**a**^				
(Mean (SD) (range)	0.16 (0.82) (0–81)	0.16 (0.82) (0–81)	0.25 (0.89) (0–15)	0.09 (0.06–0.11)
≥ 1	37,485 (8.0)	36,960 (7.9)	525 (12.5)	
**Reproductive organs (female)**^**b**^				
(Mean (SD) (range)	0.39 (1.20) (0–61)	0.39 (1.20) (0–61)	0.57 (1.44) (0–26)	0.18 (0.13–0.22)
≥ 1	90,552 (18.4)	89,598 (18.3)	954 (24.7)	
**Skin/sub cutis**				
(Mean (SD) (range)	0.64 (1.48) (0–104)	0.64 (1.48) (0–104)	0.89 (1.66) (0–24)	0.25 (0.21–0.29)
≥ 1	291,162 (30.2)	287,909 (30.1)	3253 (40.4)	
**Eye**				
(Mean (SD) (range)	0.13 (0.52) (0–49)	0.13 (0.52) (0–49)	0.22 (0.68) (0–13)	0.09 (0.07–0.10)
≥ 1	87,959 (9.1)	86,799 (9.1)	1160 (14.4)	
**Ear**				
(Mean (SD) (range)	0.20 (0.67) (0–36)	0.19 (0.65) (0–36)	1.32 (1.62) (0–25)	1.13 (1.10–1.17)
≥ 1	116,480 (12.1)	111,064 (11.6)	5416 (67.3)	
**Psychological problems**				
(Mean (SD) (range)	0.51 (1.96) (0–175)	0.50 (1.94) (0–175)	0.98 (3.23) (0–137)	0.48 (0.41–0.55)
≥ 1	145,485 (15.1)	143,470 (15.0)	2015 (25.0)	
**Social problems**				
(Mean (SD) (range)	0.11 (0.77) (0–48)	0.11 (0.77) (0–48)	0.18 (1.05) (0–30)	0.07 (0.04–0.09)
≥ 1	44,995 (4.7)	44,447 (4.7)	548 (6.8)	
**Circulatory tract**				
(Mean (SD) (range)	0.56 (1.66) (0–54)	0.56 (1.66) (0- 54)	0.88 (2.07) (0–40)	0.32 (0.28–0.37)
≥ 1	194,847 (20.2)	192,453 (20.1)	2394 (29.7)	
**Digestive tract**				
(Mean (SD) (range)	0.39 (1.41) (0–85)	0.39 (1.40)(0–85)	0.64 (1.70) (0–35)	0.24 (0.21–0.28)
≥ 1	159,997 (16.6)	157,965 (16.5)	2032 (25.2)	
**Respiratory tract**				
(Mean (SD) (range)	0.53 (1.52) (0–96)	0.53 (1.52) (0–96)	0.76 (1.77) (0–31)	0.23 (0.19–0.27)
≥ 1	227,813 (23.6)	225,262 (23.6)	2551 (31.7)	
**Urinary tract**				
(Mean (SD) (range)	0.29 (1.24) (0–68)	0.29 (1.24) (0–68)	0.36 (1.30) (0–23)	0.08 (0.05–0.11)
≥ 1	102,590 (10.6)	101,508 (10.6)	1082 (13.4)	
**Nervous system**				
(Mean (SD) (range)	0.16 (0.81) (0–75)	0.16 (0.81) (0–75)	0.30 (1.13) (0–35)	0.14 (0.11–0.16)
≥ 1	77,912 (8.1)	76,792 (8.0)	1120 (13.9)	
**Pregnancy**^**b**^				
(Mean (SD) (range)	0.24 (0.87) (0–24)	0.24 (0.87) (0–24)	0.19 (0.79) (0–11)	− 0.05 (-0.03–-0.08)
≥ 1	56,501 (11.5)	56,168 (11.5)	333 (8.6)	
**Unknown**				
(Mean (SD) (range)	0.09 (0.45) (0–32)	0.09 (0.45) ( 0–32)	0.12 (0.49) (0–10)	0.03 (0.02–0.04)
≥ 1	60,423 (6.3)	59,712 (6.2)	711 (8.8)	
**Combined ICPC codes**				
**Psychosocial**				
(Mean (SD) (range)	0.62 (2.17) (0–175)	0.61 (2.15) (0–175)	1.16 (3.47) (0–141)	0.55 (0.47–0.62)
≥ 1	175,177 (18.2)	172,846 (18.1)	2331 (29.0)	
**Other**				
(Mean (SD) (range)	5.15 (7.14) (0–229)	5.12 (7.12) (0–229)	8.60 (9.18) (0–197)	3.48 (3.28–3.68)
≥ 1	749,180 (77.7)	741,528 (77.6)	7652 (95.1)	

Psychosocial is a combination of ICPC codes: psychological problems and social problems. Other is the combination of all other ICPC codes (exception psychological problems and social problems).

^a^Only calculated for males.

^b^Only calculated for females.

Compared to those without tinnitus, patients with tinnitus were more likely to have one or more primary healthcare contacts, independent of age group and gender [OR 5.71 (95% CI 5.14–6.35)] (Table 3).

**Table 3:** Results of the logitisic regressions corrected for age and gender.

Outcome	N	Predictors		Odds ratio 95% CI
≥ 1 contact	963,880	Tinnitus	Yes	5.71 (5.14–6.35)
			No	Ref
≥ 1 referral	404,975^a^	Tinnitus	Yes	2.67 (2.49–2.86)
			No	Ref
≥ 1 prescription	963,880	Tinnitus	Yes	2.29 (2.15–2.45)
			No	Ref

^a^Perfomed in patients for whom referral data was available (404,975).

### Number of referrals to medical specialists

For 401.572 of 955,830 people without tinnitus (42.0%), referral data was available. 98.326 of 401.572 (24.5%) people without tinnitus had one or more referrals to medical specialist. Referral data was available for 3403 of 8050 (42.3%) patients with tinnitus. 1597 of 3403 patients with tinnitus (46.9%) had one or more referrals to a medical specialist. (Table 4.) Compared to those without tinnitus, patients with tinnitus were more likely to have one or more referrals, independent of age group and gender(OR 2.67 (2.49–2.86) (Table 3).

**Table 4:** Number of referrals and total number of drug prescriptions of patients with and without tinnitus drug.

	Total	Tinnitus		Mean difference (95% confidence interval)
		No	Yes	
Referrals^a^ (mean (SD) range)	0.32 (0.63) (0–8)	0.31 (0.63) (0–8)	0.68 (0.90) (0–8)	0.37 (0.34–0.40)
***N *(%)**				
0	305,052 (75.3)	303,246 (75.5)	1806 (53.1)	
≥ 1	99,923 (24.7)	98,326 (24.5)	1597 (46.9)	
**Elaboration of ≥ 1 referral (n (%))**^**a**^				
1	77,276 (19.1)	76,203 (19.0)	1073 (31.5)	
2	17,875 (4.4)	17,503 (4.4)	372 (10.9)	
3	3374 (0.9)	3665 (0.9)	109 (3.2)	
4	792 (0.2)	759 (0.2)	33 (1.0)	
5	157 (0.0)	149 (0.0)	8 (0.2)	
6	39 (0.0)	38 (0.0)	1 (0.0)	
7	7 (0.0)	7 (0.0)	0 (0.0)	
8	3 (0.0)	2 (0.0)	1 (0.0)	
Drug prescriptions (mean (SD))	3.41 (3.95) (0–42)	3.4 (3.94) (0–42)	4.62 (4.31) (0–40)	1.2 (1.13–1.31)
≥ 1	715,457 (74.2)	708,453 (74.1)	7004 (87.0)	

^a^Assessed in those for whom referral data was available (n = 404,975), available for 401.572 people without tinnitus and 3403 people with tinnitus.

### Numbers of prescriptions

For the complete sample a mean of 3.4 (SD 3.9) drug prescriptions were registered in 2018 (range 0–42). 715,457 (74.2% of 963,880) had one or more prescriptions in 2018. Patients with tinnitus had 1.2 (mean difference, 95% CI) 1.13–1.31) more drug prescriptions than people without tinnitus (Table 4). Compared to those without tinnitus, patients with tinnitus were more likely to have one or more precreptions,independent of age group and gender. (OR 2.29 (2.15–2.45)) (Table 3).

## Discussion

In this study we assessed the differences in primary health care consumption between patients with tinnitus and people without tinnitus in a Dutch cohort. 0.8% of the patients (8050 out of 963,880) had an open tinnitus episode and were therefore defined as a patient with tinnitus. Patients with tinnitus used more primary care consultations compared to people without tinnitus. Patients with tinnitus were more often referred to medical specialists compared to people without tinnitus, and patients with tinnitus were more often prescribed drugs.

We observed four more (mean difference) primary care consultations in patients with tinnitus compared to people without tinnitus. This is a larger difference than the results of a study on health care utilization in United States veterans^27^. Even though they identified a higher total healthcare usage (including medical specialist care), they only found a mean of 2.9 visits to primary health care for those with tinnitus, compared to a mean of 2.2 visits for veterans without tinnitus over a five year period^27^. This difference might be explained by the differences in health care systems or studied populations. Next, we find more annual GP visits for patients with tinnitus compared to a Dutch study by Maes et al. out of 2013^13^. They describe a mean of 7.78 contacts annually to the GP for tinnitus-related health care in people with tinnitus. This could be explained by the fact that their study was based on a sample that was referred to (and in need of) specialist tinnitus care. Moreover, we found that 0.8% of all patients that visited the GP in 2018 had an open tinnitus episode. This is different to the prevalence numbers described in the study be Maes et al.. They based their results on the assumption that 30% of individuals would experience tinnitus at some point in their life, and 10% would require medical help^13,28,29^. Apart from the differences in population, another explanation of the large variance could be the use of different definitions to determine tinnitus prevalence numbers^2^.

In our study consultations for all organ systems (except pregnancy) were more frequent in patients with tinnitus. Only consultations in the ICPC chapter “Ears’’ showed a clinically relevant difference. This could be related to the tinnitus consultation itself which is coded under this ICPC chapter or to hearing problems, which is one of the most important risk factors for tinnitus. Moreover, in our study we found a difference between those with and without tinnitus in number of consultations for psychological problems (mean difference in visits between groups 0.48) and musculoskeletal problems (mean difference in visits between groups 0.44). This is not surprising considering the fact that psychological problems such as anxiety, depression or sleep- and concentration difficulties are common comorbidities of tinnitus^30^. An increase in musculoskeletal problems in tinnitus patients might be explained by commonly described comorbidities such as temporomandibular dysfunction (TMD)^17,31^. In a systematic review from 2019 the prevalence of tinnitus in patients with temporomandibular dysfunction ranged between 3.7 and 70%^32^. Also a relatively large component of the difference in number of consultations between both groups was found in the circulatory tract ICPC chapter (mean difference 0.32). Cardiovascular diseases, such as high blood pressure have been described to be associated with tinnitus. Cardiovascular diseases are believed to damage inner ear circulation, and consequently cause tinnitus^17,33^.

Next, we noticed a higher number of referrals to medical specialists. 46.9% of patients with tinnitus were referred to a medical specialist at least once in 2018, compared to 24.5% of the people without tinnitus. Our data did not include information about which medical specialists were consulted. Whether the higher number of referrals can be explained by the bothersome nature of the experienced tinnitus cannot be concluded by the presented data. As a first step in tinnitus health care the severity of the tinnitus should be assessed by the primary health care provider. Only, when the tinnitus is bothersome a referral to an otorhinolaryngologists or an audiologist is indicated as described in the European guideline^10^.

Patients with tinnitus received a mean of 1.2 more drug prescriptions in 2018, compared to people without tinnitus. We did not have data about which drugs were prescribed. This is of interest since pharmaceutical treatment for tinnitus is not recommended, because of the lack of effectiveness in reducing tinnitus symptoms. However, medication is still prescribed in clinical practice^34^. In a previous study it was estimated that doctors write over 4 million off-label prescriptions annually in Europe and the United States for the relief of tinnitus^35^. These include anti-depressives, prednisolone, betahistine and anti-epileptic drugs^36^. The higher number of drug prescriptions in our data could suggest that these prescriptions are not only because of the tinnitus, but are more likely related to other morbidities of patients with tinnitus.

### Strengths and limitations

The large cohort of participants to the primary care database is a representative sample of the Dutch population^20,37^. This provides unique insight in primary health care usage of those with tinnitus compared to those without. One of the limitations is our definition of a tinnitus patient. A person was defined as a tinnitus patient when they had an open episode of tinnitus in 2018. This was defined as any registration of tinnitus in the patient’s electronic health record within the period mid-2017 to the end of 2018^23^. Next, there might be those that experience or suffer from tinnitus in the group “people without tinnitus”. They could have visited the GP for tinnitus in previous years or not at all. Also, we are not certain that the patients with tinnitus actually consulted the GP for the tinnitus. Next, there might be variability in the registered tinnitus diagnoses. It might differ per practice whether tinnitus was registered, some GPs might not register the tinnitus if a person contacts the GP for other reasons. Also, our study is limited to information about the number of contacts, referrals and prescriptions.) Since this study had an explorative nature, it does not entail information on the reason for referrals, to which medical specialists patients were referred or which drugs were prescribed.

In our study we demonstrated a higher number of consultations in primary care in patients with tinnitus compared to people without tinnitus. This might be the result of the fact that patients with tinnitus are less healthy or have more mental or physical complaints compared to people without tinnitus. A combination of both might also be possible, or it could be neither. This cross-sectional study does not inform us about the etiological relationship between tinnitus and co-morbidities. Large observational studies could help explore this causal relationship, which could contribute in the search for interventions. Also, a more specified analysis on the amount of visits for different comorbidities (tinnitus related or unrelated), rather than an umbrella term used in this study, for more in-depth knowledge. This would be similar to a Nivel primary care database study that looked at specific diagnoses codes in relation to inflammatory arthritis^38^.

## Conclusion

We conclude that patients with tinnitus had more primary health care contacts compared to people without tinnitus, with an average of four more primary care consultations in one year. Patients with tinnitus received more drug prescriptions and were more frequently referred to the medical specialists.

## Data Availability

The data that support the findings of this study are available from Nivel but restrictions apply to the availability of these data, which were used under license for the current study, and so are not publicly available. According to the Governance of Nivel Primary Care Database, the Steering committee with representatives from national associations of general practitioners decide about the use of the data. Therefore, permission from this Steering committee is required for each request to do research with the data. Data are available upon request (Zorgregistraties@nivel.nl).
